# Mental health and post-traumatic stress among unprivileged people in the aftermath of COVID-19 pandemic in Southwest Bangladesh: a cross-sectional study

**DOI:** 10.1080/28324765.2025.2484006

**Published:** 2025-03-26

**Authors:** Md. Salauddin Khan, Maliha Mahazabin, Ishita Shahid Sams, Lasker Ershad Ali, Umama Khan

**Affiliations:** a Statistics Discipline, Science Engineering and Technology School, Khulna University, Khulna, Bangladesh; b Department of Sociology, Government Brajalal College, Khulna, Bangladesh; c Mathematics Discipline, Science Engineering and Technology School, Khulna University, Khulna, Bangladesh; d Department of Genetic Engineering and Biotechnology, Jashore University of Science and Technology, Jashore, Bangladesh

**Keywords:** Marginalized, post-traumatic stress disorder, Bangladesh, COVID-19, Khulna City, slum dwellers

## Abstract

The COVID-19 pandemic has adversely influenced the mental health of individuals in developing countries, whereas the impoverished urban population has been affected the most due to unattainable mental healthcare and the societal stigma surrounding mental illness. This research aims to assess the mental health status of slum dwellers in the aftermath of COVID-19. Psychological well-being, post-traumatic stress disorder (PTSD), anxiety and depression symptoms were evaluated through structured face-to-face interviews with 404 individuals from July to September 2023. More than half of the participants experienced poor mental well-being, while women tended to have high symptoms of depression (35%) and PTSD (18%). Slum dwellers who experienced food scarcity and used unimproved or community latrines during the pandemic were more likely to report symptoms of PTSD. Furthermore, those who experienced any crime or domestic violence (OR = 2.19; 95% CI: 1.08–4.45) and lost their jobs (OR = 1.56; 95% CI: 1.04–2.33) during the pandemic were more likely to report poor mental health. Therefore, the vulnerable population urgently needs targeted interventions, such as skill-building opportunities for the unemployed, gender-segregated sanitation facilities and training programs to support their mental health. Prioritizing mental health awareness campaigns and establishing strong social safety nets can help communities become more resilient to future pandemics and crises.

## 1. Introduction

Mental wellness is a fundamental pillar of overall well-being, intrinsically linked to one’s ability to maintain an active and profound life (Galderisi et al., 2015). Since the outbreak of COVID-19, the pandemic has triggered an unprecedented global crisis, affected all aspects of human life, and threatened physical as well as psychological well-being (Jain et al., 2020). As the virus spreads, vulnerable populations in urban settings—particularly individuals in overcrowded informal settlements with limited access to essential services, such as slum dwellers (Corburn & Sverdlik, 2019; Olotuah, 2012)—faced unique and acute challenges. Among the myriad adversities faced by slum dwellers, their mental health status remained an often neglected yet critical concern (Nejad et al., 2021).

The COVID-19 pandemic imposed tremendous pressure on global healthcare facilities, amplifying pre-existing disparities in access to mental healthcare (Luo et al., 2020; Yu et al., 2020). Previous pandemics, such as the SARS outbreak in 2003 and the H1N1 influenza pandemic in 2009, emphasized the possible long-term psychological consequences for infected and non-infected populations (Chau et al., 2021; Cullen et al., 2020). Similar patterns of poor mental health and post-traumatic stress disorder (PTSD) symptoms were observed among the general population during the COVID-19 pandemic (Boyraz & Legros, 2020; Paul et al., 2021). Prolonged lockdowns and living in isolation may yield numerous adverse consequences, as empirical findings revealed that at least one out of every five people experienced clinically significant mental health issues during the pandemic (Helter et al., 2022; Jain et al., 2020). Longitudinal studies have found that the number of cases of anxiety and depression increased significantly among the general people (notably women, young individuals and those with preschool-aged children) in the UK and China amid the COVID-19 pandemic (Pierce et al., 2020; Wang et al., 2020). Fear of infection, loss of income, social isolation and uncertainty about the future are just a few of the stressors the pandemic has brought, all of which can negatively influence mental health (Holmes et al., 2020). It is crucial to focus on reaching out to vulnerable groups, including the elderly, undocumented immigrants, homeless individuals and those with mental health problems, to ensure their well-being during these difficult times (Galea et al., 2020). Prior studies revealed that individuals who had recovered from COVID-19 experienced symptoms of PTSD and struggled with a lack of social aid during the recovery period (Sujan et al., 2023). Adequate social support is a protective factor for preventing the development of mental health issues while coping with stress. Support from others can also help alleviate life stressors, especially for those experiencing higher stress levels (Yu et al., 2020).

In many South Asian countries, including Bangladesh, mental health is rarely prioritized as a health policy strategy, and numerous sociocultural barriers, as well as the stigma of mental disorders, can discourage people from opening up about their struggles (Hossain et al., 2019, 2020). In the context of the COVID-19 pandemic, there is anticipated to be a considerable surge in substance abuse, poor mental health, loneliness and domestic violence in Bangladesh (Nabila Ashraf et al., 2021, Yasmin et al., 2023). Prior studies revealed that the prevalence of stress increased by 76.6% among low-income people during the pandemic (Paul et al., 2021), and the effects may be even worse among slum residents, considering their deprived situations. The loss of income and employment opportunities as a consequence of lockdowns and restrictions has increased financial stress and uncertainty, exacerbating mental health concerns (Khan et al., 2024; Paul et al., 2021). In addition, the overcrowded living conditions prevalent in slums increase the risk of virus transmission and elevate anxiety levels (Koly et al., 2021). Experiencing the devastating consequences of the pandemic and uncertain prospects can potentially result in PTSD and even suicidal thoughts and actions (Chatterjee et al., 2020). Individuals with a low level of education, a lack of support from relatives, those who have lost family members and friends, difficulty affording medications and long COVID-19 symptoms increase the risk of developing PTSD (Soloveva et al., 2021; Vo et al., 2024; Zeng et al., 2022). Notably, COVID-19-related aspects such as residing in the most affected areas, history of infection, financial difficulties and loss of employment lead to suicidal ideation (Gunnell et al., 2020; Shi et al., 2021). The stigma towards mental health further impedes help-seeking behavior, making it difficult for slum dwellers to seek and receive appropriate support (Rabbani et al., 2018). Unfortunately, such circumstances can exacerbate adverse mental health outcomes and block the potential for timely intervention.

The mental health repercussions of the COVID-19 pandemic are multifaceted. The pandemic has disproportionately affected economically disadvantaged groups, and slum dwellers are no exception. While several studies have been done on the psychological well-being of various communities, there is still no research on the mental health of individuals residing in Khulna City slums in the aftermath of COVID-19. Khulna in southern Bangladesh is a climate hotspot where most slum dwellers have moved due to frequent climate-induced disasters. Regrettably, nearly one-third of coastal communities in Bangladesh experience the adverse effects of natural disasters annually (Parvin et al., 2023). The particular socioeconomic setting of these communities requires an appropriate study to identify their mental health concerns and provide solutions to meet their specific needs. Our research aims to assess the psychological conditions among Khulna City slum dwellers in the aftermath of the COVID-19 pandemic. By conducting a comprehensive survey and using standardized psychological assessment tools, we aim to quantify anxiety and depression symptoms and evaluate the associated demographic, socioeconomic, surrounding environment, health and COVID-19 related factors of mental well-being and PTSD symptoms prevalent in this population.

## 2. Materials and methods

### 2.1. Design and participants

A cross-sectional study was conducted among the residents of 15 slums in Khulna City Corporation (KCC) from July to September 2023. The inclusion criteria were ’participants residing in the slum during the outbreak of COVID-19’, ’participants who were over 16 years of age’ and ’participants who expressed a willingness to respond to the survey’, while the standard for exclusion was ’participants who were over 70 years of age’. Eight graduate-level research assistants were selected and trained for data collection and utilized a face-to-face survey methodology to engage participants in the slum area. We employed the online survey tool ’Kobo Toolbox’ to collect the data and the survey questionnaire was uploaded to each data collector’s mobile phone via the Kobo Toolbox application. A structured questionnaire in English was created using the Kobo Toolbox and then transformed into Bangla with a forward-backward translation method for carrying out the survey among slum residents. The questionnaire was organized into four sections: (1) Socio-demographic and pandemic-related characteristics; (2) Mental well-being; (3) Anxiety and depression over the past 2 weeks; (4) Post-traumatic stress related to pandemic. Using a two-stage sampling method, we determined the number of slums to select from each ward and collected data from 404 households (one eligible participant was selected from each household). First, we calculated that 404 participants were required to reach a 5% level of precision using Cochran’s formula. Subsequently, the sample households for each slum were obtained by employing probability proportional to size (PPS) sampling, with the sample size for each chosen slum obtained through its proportion to the overall slum population. From each selected slum, a representative sample of slum dwellers was randomly selected to ensure the inclusivity and generalizability of the findings. The required sample sizes were estimated following Cochran's formula, where standard normal value = 0.05 (95% confidence level), 0.05 (error of margin), and the percentage of urban slum dwellers who experienced psychological illness in the aftermath of COVID-19, which was identified from the pilot survey to be around 48%. Finally, considering 5% non-response, the total sample size was estimated. Of the 404 participants, most slum dwellers had primary education (51.24%) and 28.22% of slum dwellers had no formal education. Around 20% of slum dwellers felt unsafe in their neighborhood and 29.95% had a chronically ill family member. Only 9.9% migrated to their home village due to the COVID-19 pandemic. Most slum dwellers were unable to access healthcare due to financial constraints (62.38%) and borrowed money to meet their daily necessities (70.54%) during the pandemic.

This study was approved by Khulna University Ethical Clearance Committee (Reference No. KUECC-2023-07-40) review board, and all procedures were conducted in accordance with the relevant guidelines and regulations. Informed consent was obtained from all participants prior to their inclusion in the study. All respondents were informed about their role as participants, as well as the anonymity, confidentiality of their responses, and the right to withdraw from the interview at any moment if they were unwilling to participate.

### 2.2. Study measure

#### 2.2.1. WHO-5

The study assessed the mental well-being of slum dwellers using a validated psychological assessment tool: the ‘World Health Organization-Five Mental Well-being Index (WHO-5)’ questionnaire. WHO-5 is a well-accepted and reliable way to evaluate current psychological well-being. Scores range from 0 to 100; a score of 50 indicates poor well-being (Omani-Samani et al., 2019; Topp et al., 2015). This scale has previously been utilized in research conducted among slum dwellers in Bangladesh (Gruebner et al., 2012). In this study, the Cronbach’s alpha value for ‘WHO-5’ was 0.779, indicating a satisfactory internal consistency (Crocker & Algina, 1986). For analysis purposes, the outcome variable ‘WHO-5’ was categorized in binary response: ’poor mental well-being’ coded as ’1’ and ’good mental well-being’ coded as ’0’.

#### 2.2.2. PTSD

The Bangla adaptation of the five-item Primary Care PTSD Screen for DSM-5 used to detect PTSD symptoms (Cheng et al., 2021; Prins et al., 2016) which assesses signs of traumatic stress disorder within the last month through five queries that necessitate a binary response to relive the event, avoiding reminders, physical reactions, emotional detachment and altered thoughts of guilt and blame stemming from the trauma. Before enquiring about five PTSD symptoms, we first asked the respondents, ’Since the beginning of COVID-19, have you ever felt frightened, horrible or traumatic caused by COVID-19 or related factors?’ If the response was yes, we posed five questions about the COVID-19 pandemic as a traumatic event to identify PTSD symptoms. A forward-backward translation method was utilized to maintain the validity of the questionnaire. This scale has previously been utilized in research conducted in Bangladesh (Alam et al., 2022). Cronbach’s alpha value for ’PTSD’ was 0.832, indicating greater consistency. Primary care PTSD scores are measured on a scale of 1 to 5, and if the overall score is 3, this suggests a clinically significant level of PTSD symptoms (Sun et al., 2024). For analysis purposes, the outcome variable PTSD symptom was categorized in binary response; the presence of PTSD symptoms was coded as ’1’, and the absence of the symptoms was coded as ’0’.

#### 2.2.3. Patient health questionnaire-4

PHQ-4 is a four-item Likert scale question used to assess anxiety and depression symptoms over the past 2 weeks (Kroenke et al., 2009; Stanhope, 2016). The total score of the PHQ-4 ranges from 0 to 12, classified into four categories: None (0–2), Mild (3–5), Moderate (6–8) and Severe (9–12). The initial two items of the PHQ-4 (known as GAD-2) were used to evaluate anxiety symptoms (Hlynsson & Carlbring, 2024; Hughes et al., 2018), while the last two items (known as PHQ-2) were used to evaluate depression symptoms (Kroenke et al., 2003; Löwe et al., 2005). Scores range from 0 to 6 for each subscale; a score of ≥3 indicates the presence of the symptoms. In this study, the Cronbach’s alpha values for PHQ-4 were 0.726. The PHQ-4 was employed to assess the rate of anxiety and depression symptoms among slum dwellers.

#### 2.2.4. Demographic, socioeconomic, surrounding environment, health and COVID-19-related factors

Participants’ socioeconomic status was assessed using Kuppuswamy’s Socioeconomic Scale (SES) based on the head of the household’s information (Bairwa et al., 2013; Satter et al., 2022). According to the measure, the socioeconomic status was categorized into three groups: ’extreme poor or below the poverty line’, ’poor’ and ’lower middle’ group. Demographic, socioeconomic, surrounding environment, health and COVID-19-related factors were presented in supplementary file 2. All predictor variables were selected for our models based on previously published research on mental well-being and PTSD in the context of the COVID-19 pandemic (Akter et al., 2021; D’alessandro et al., 2020; Islam et al., 2021; Long et al., 2022; Luthra et al., 2023; Mouratidis, 2020; Zhang et al., 2020).

#### 2.2.5. Statistical analysis

To determine the post-COVID mental health situation of slum dwellers, binary logistic regression was applied (Pituch & Stevens, 2015). A bivariate binary logistic model was performed to identify the factors linked to the outcome variables (’WHO-5 mental well-being’ and ’PTSD’). After performing binary logistic regression (unadjusted model), variables with a p-value <0.25 were selected for multivariable regression (adjusted model) to determine the presence and severity of the predictor and outcome variables (Agresti, 2002). Before performing the logistic regression, we checked for multicollinearity in the model. Our model demonstrated the absence of multicollinearity, as none of the variables had a variance inflation factor (VIF) score higher than 2.5 or a tolerance value lower than 0.1 (Kim, 2019). Subsequently, the adequacy of the model was tested using the Hosmer and Lemeshow goodness-of-fit test. The results demonstrated p-values of 0.628 and 0.853 for ’PTSD’ and ’mental well-being’ models, respectively, indicating that the data fit the models well. Moreover, the areas under the ROC curves for ’PTSD’ and ’mental well-being’ were 0.82 and 0.79 (supplementary file 3), which implies that the logistic regression models were well fitted. The data analysis was performed utilizing the STATA software, with a significance threshold set at *p* < 0.05 with a 95% confidence interval.

## 3. Results

### 3.1. Prevalence of anxiety, depression, PTSD symptoms and mental well-being

**Figure 1.**
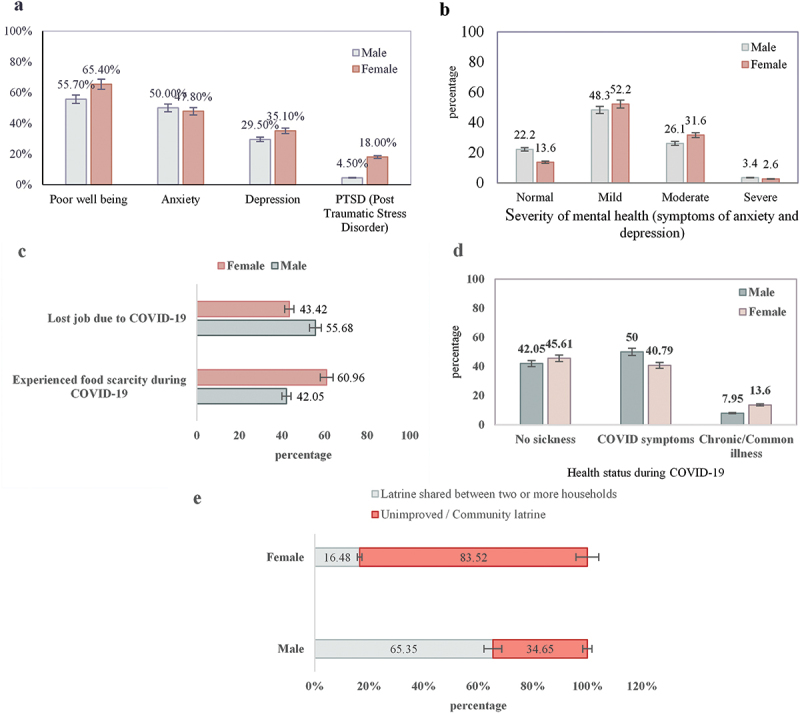
(a) Mental health hazard stratification of gender in the aftermath of COVID-19, (b) PHQ-4 (anxiety and depression symptoms), (c) job loss and food scarcity status during COVID-19, (d) self-reported health status during COVID-19 and (e) sanitation facility among individuals living in slum areas.

The findings of our study indicate that men (50%) reported slightly higher levels of anxiety symptoms compared to women (47.8%), while women tended to experience higher levels of depression (35.10%), poor mental well-being (65.40%) and PTSD symptoms (18%) in the aftermath of COVID-19 (Figure 1). The graph indicates that more than 52% of the women and 48% of the men experience mild anxiety and depression symptoms.

### 3.2. Multivariate analysis

#### 3.2.1. Mental well-being

Table 1 demonstrates the findings of logistic regression with odds ratio (OR1) and adjusted odds ratio (AOR1) models of mental well-being among Khulna city slum dwellers. Our study revealed that women were more likely to experience poor mental health (AOR1 = 2.85; 95% CI: [1.71–4.76]; p < 0.001) than men, and slum dwellers aged 26–40 years (AOR1 = 1.96; 95% CI: [1.07–3.62]; p = 0.030) and older than 40 years (AOR1 = 2.13; 95% CI: [1.01–4.46]; p = 0.046) were more likely to experience poor mental well-being than those aged 25 years or less. Participants who lived in the slum area for 16–30 years (AOR1 = 1.85; 95% CI: [1.03–3.33]; p = 0.040) and more than 30 years (AOR1 = 2.02; 95% CI: [1.04–3.92]; p = 0.037) were more prone to have poor mental health than those who lived in the slum area for 15 years or less. Moreover, slum dwellers who lived with four to five family members (AOR1 = 1.78; 95% CI: [1.02–3.13]; p = 0.042) were more likely to report poor mental well-being compared to those who had up to three family members.

**Table 1.** Association of socio-demographic and other factors with the current mental well-being of urban slum dwellers of the consequence of COVID-19

Variable	Categories	World Health Organization-Five Mental Well-being Index (WHO-5)						
		Good mental well-being		Poor mental well-being				
		n (%)		n (%)	OR1 (95% CI)	P-value	AOR1 (95% CI)	P-value
Gender	Male	78 (44.3)		98 (55.7)	Ref.		Ref.	
	Female	79 (34.6)		149 (65.4)	1.50 (1.003–2.25)	0.048*	2.85 (1.71–4.76)	<0.001*
Religion	Other Religion	7 (30.4)		16 (69.6)	Ref.			
	Muslim	150 (39.4)		231 (60.6)	0.67 (0.27–1.68)	0.396	_	
Age (years)	25	54 (56.3)		42 (43.8)	Ref.		Ref.	
	26–40	56 (32.2)		118 (67.8)	2.71 (1.62–4.53)	<0.001*	1.96 (1.07–3.62)	0.030*
	40	47 (35.1)		87 (64.9)	2.37 (1.39–4.07)	0.002*	2.13 (1.01–4.46)	0.046*
Slum living year	15	50 (49.0)		52 (51.0)	Ref.		Ref.	
	16–30	64 (40.0)		96 (60.0)	1.44 (0.87–2.38)	0.152	1.85 (1.03–3.33)	0.040*
	30	43 (30.3)		99 (69.7)	2.21 (1.31–3.75)	0.003*	2.02 (1.04–3.92)	0.037*
Monthly household income (BDT	7000	32 (41.0)		46 (59.0)	Ref.			
	7001–12000	54 (36.5)		94 (63.5)	1.21 (0.69–2.12)	0.504	_	
	12000	71 (39.9)		107 (60.1)	1.05 (0.61–1.80)	0.864		
Education level	No formal education	40 (35.1)		74 (64.9)	Ref.			
	Primary	84 (40.6)		123 (59.4)	0.79 (0.49–1.27)	0.334	_	
	Secondary/Higher	33 (39.8)		50 (60.2)	0.82 (0.46–1.47)	0.503		
Family size		53 (50.5)		52 (49.5)	Ref.		Ref.	
	4–5	69 (35.0)		128 (65.0)	1.89 (1.17–3.06)	0.010*	1.78 (1.02–3.13)	0.042*
		35 (34.3)		67 (65.7)	1.95 (1.11–3.41)	0.019*	1.51 (0.79–2.13)	0.213
Family member suffering from chronic illness^a^	No	130 (45.9)		153 (54.1)	Ref.		Ref.	
	Yes	27 (22.3)		94 (77.7)	2.96 (1.82–4.82)	<0.001*	3.14 (1.76–5.58)	<0.001*
SES^b^ (Socioeconomic Status)	Extreme Poor	2 (25.0)		6 (75.0)	Ref.			
	Poor	109 (37.3)		183 (62.7)	0.56 (0.11–2.82)	0.482	_	
	Lower middle class	46 (44.2)		58 (55.8)	0.42 (0.08–2.18)	0.302		
Sanitation facility during Pandemic	Latrine shared between two or more households/Family latrine	87 (38.5)		139 (61.5)	Ref.			
	Unimproved/Community latrine	70 (39.3)		108 (60.7)	0.96 (0.64–1.44)	0.865	_	
Garbage disposal	No fixed place	60 (36.8)		103 (63.2)	Ref.			
	Fixed place/City corporation dustbin	97 (40.2)		144 (59.8)	0.86 (0.57–1.30)	0.487	_	
Health status during COVID	Chronic/Common illness	13 (28.9)		32 (71.1)	Ref.		Ref.	
	COVID Symptoms	63 (34.8)		118 (65.2)	0.76 (0.37–1.55)	0.453	0.71 (0.29–1.69)	0.439
	No Sickness	81 (45.5)		97 (54.5)	0.49 (0.24–0.98)	0.046*	0.57 (0.24–1.34)	0.197
Daily sleep hours	Less than 7 h	80 (35.1)		148 (64.9)	Ref.		Ref.	
	7–9 h	66 (42.6)		89 (57.4)	0.73 (0.48–1.11)	0.139	1.18 (0.72–1.95)	0.513
	More than 9 h	11 (52.4)		10 (47.6)	0.49 (0.20–1.21)	0.121	1.29 (0.43–3.88)	0.650
Experienced food scarcity due to COVID-19	No	74 (38.7)		117 (61.3)	Ref.			
	Yes	83 (39.0)		130 (61.0)	0.99 (0.66–1.47)	0.963	_	
Experienced any crime or domestic violence during the pandemic	No	146 (40.8)		212 (59.2)	Ref.		Ref.	
	Yes	11 (23.9)		35 (76.1)	2.19 (1.08–4.45)	0.030*	1.12 (0.48–2.57)	0.79
Migrate or move to the home village during the pandemic	No	144 (39.6)		220 (60.4)	Ref.			
	Yes	13 (32.5)		27 (67.5)	1.36 (0.68–2.72)	0.386	_	
Have you received any support during the pandemic	No	80 (35.1)		148 (64.9)	Ref.		Ref.	
	Yes	77 (43.8)		99 (56.2)	0.69 (0.46–1.04)	0.077	0.71 (0.44–1.15)	0.171
Perception of safety in the neighborhood	Safe	147 (45.7)		175 (54.3)	Ref.		Ref.	
	Unsafe	10 (12.2)		72 (87.8)	5.04 (3.01–10.14)	<0.001*	6.79 (3.94–11.25)	<0.001*
Employment situation during the pandemic	No change	91 (44.0)		116 (56.0)	Ref.		Ref.	
	Lost job/working hours reduced	66 (33.5)		131 (66.5)	1.56 (1.04–2.33)	0.032*	1.29 (0.71–2.35)	0.395
Unable to seek healthcare due to financial difficulties during pandemic	No	68 (44.7)		84 (55.3)	Ref.		Ref.	
	Yes	89 (35.3)		163 (64.7)	1.48 (0.98–2.24)	0.060	0.65 (0.36–1.17)	0.152
Household income status during the pandemic	Stayed the same	53 (52.5)		48 (47.5)	Ref.		Ref.	
	Decreased	104 (34.3)		199 (65.7)	2.11 (1.34–3.34)	0.001*	1.55 (0.81–2.97)	0.181
Borrow money to meet daily needs during pandemic	No	63 (52.9)		56 (47.1)	Ref.		Ref.	
	Yes	94 (33.0)		191 (67.0)	2.28 (1.48–3.54)	<0.001*	1.96 (1.09–3.51)	0.024*

*Significant at *p* < 0.05; ^CI^Confidence Interval, ^AOR^Adjusted Odds Ratio; ^OR^Odds Ratio; ^a^chronic illness such as cancer, heart disease, stroke, diabetes, kidney failure, arthritis; ^b^Modified Kuppuswamy Socioeconomic scale.

Slum dwellers who had a family member with a chronic illness (AOR1 = 3.14; 95% CI: [1.76–5.58]; *p* < 0.001) were three times more prone to report poor mental health than those who did not have a chronically ill family member. Moreover, slum dwellers who felt unsafe in their neighborhood (AOR1 = 6.79; 95% CI: [3.94–11.25]; *p* < 0.001) were more likely to have poor mental well-being than those who felt safe.

During COVID-19, participants with chronic or common illness were more likely to experience poor mental well-being than those with no sickness (OR1 = 0.49; 95% CI: [0.24–0.98]; *p* = 0.046). Additionally, those who experienced any crime or domestic violence during the pandemic were two times more likely (OR1 = 2.19; 95% CI: [1.08–4.45]; *p* = 0.030), and those who lost their job were 56% more likely (OR1 = 1.56; 95% CI: [1.04–2.33]; *p* = 0.032) to experience poor mental health, even after the pandemic. Participants who borrowed money to meet daily needs during the pandemic (AOR1 = 1.96; 95% CI: [1.09–3.51]; *p* = 0.024) were more likely to have poor mental well-being.

#### 3.2.2. PTSD symptoms

The findings from the multivariate analysis in Table 2 show a statistically significant association between gender and PTSD symptoms, implying that female slum dwellers (AOR2 = 3.16; 95% CI: [1.22–8.19]; p = 0.018) were three times more prone to experience PTSD symptoms than males in the aftermath of the pandemic. Slum dwellers who completed secondary or higher education (OR2 = 0.36; 95% CI: [0.14–0.95]; p = 0.040) were less likely to report PTSD symptoms in the aftermath of COVID-19 than those with no formal education.

**Table 2.** Association of socio-demographic and other factors with the PTSD symptoms of urban slum dwellers

Variable	Categories	PTSD (post-traumatic stress disorder)					
		No (%)	Yes (%)	OR2 (95% CI)	P-value	AOR2 (95% CI)	P-value
Gender	Male	168 (95.5)	8 (4.5)	Ref.		Ref.	
	Female	187 (82.0)	41 (18.0)	4.60 (2.09–10.10)	<0.001*	3.16 (1.22–8.19)	0.018*
Religion	Other Religion	15 (65.2)	8 (34.8)	Ref.		Ref.	
	Muslim	340 (89.2)	41 (10.8)	0.23 (0.09–0.57)	0.001*	0.16 (0.05–0.50)	0.002*
Age (years)	25	84 (87.5)	12 (12.5)	Ref.			
	26–40	148 (85.1)	26 (14.9)	1.23 (0.59–2.56)	0.581	_	
	40	123 (91.8)	11 (8.2)	0.62 (0.26–1.48)	0.288		
Slum living year		88 (86.3)	14 (13.7)	Ref.			
	16–30	141 (88.1)	19 (11.9)	0.85 (0.40–1.77)	0.660	_	
	30	126 (88.7)	16 (11.3)	0.79 (0.37–1.71)	0.565		
Monthly household income (BDT)	7000	60 (76.9)	18 (23.1)	Ref.		Ref.	
	7001–12000	133 (89.9)	15 (10.1)	0.38 (0.18–0.79)	0.011*	0.55 (0.23–1.34)	0.188
		162 (91.0)	16 (9.0)	0.33 (0.16–0.68)	0.003*	0.73 (0.26–2.08)	0.561
Education level	No formal education	94 (82.5)	20 (17.5)	Ref.		Ref.	
	Primary	184 (88.9)	23 (11.1)	0.58 (0.31–1.12)	0.108	0.83 (0.39–1.74)	0.619
	Secondary/Higher	77 (92.8)	6 (7.2)	0.36 (0.14–0.95)	0.040*	0.84 (0.26–2.78)	0.780
Family size		91 (86.7)	14 (13.3)	Ref.			
	4–5	172 (87.3)	25 (12.7)	0.94 (0.46–1.90)	0.874	_	
	5	92 (90.2)	10 (9.8)	0.71 (0.29–1.67)	0.429		
Family member suffering from chronic illness^a^	No	244 (86.2)	39 (13.8)	Ref.		Ref.	
	Yes	111 (91.7)	10 (8.3)	0.56 (0.27–1.17)	0.124	0.65 (0.28–1.53)	0.325
SES^b^ (Socioeconomic Status)	Extreme Poor	5 (62.5)	3 (37.5)	Ref.		Ref.	
	Poor	255 (87.3)	37 (12.7)	0.24 (0.06–1.05)	0.059	0.27 (0.05–1.60)	0.151
	Lower middle class	95 (91.3)	9 (8.7)	0.16 (0.03–0.77)	0.023*	0.42 (0.05–3.32)	0.411
Sanitation facility during Pandemic	Latrine shared between two or more households/Family latrine	142 (79.8)	36 (20.2)	Ref.		Ref.	
	Unimproved/Community latrine	213 (94.2)	13 (5.8)	4.15 (2.13–8.11)	<0.001*	1.82 (0.76–4.36)	0.179
Garbage disposal	No fixed place	139 (85.3)	24 (14.7)	Ref.		Ref.	
	Fixed place/City corporation dustbin	216 (89.6)	25 (10.4)	0.67 (0.37–1.22)	0.191	1.07 (0.52–2.20)	0.853
Health status during COVID	Chronic/Common illness	38 (84.4)	7 (15.6)	Ref.			
	COVID symptoms	156 (86.2)	25 (13.8)	0.87 (0.35–2.16)	0.764	_	
	No sickness	161 (90.4)	17 (9.6)	0.57 (0.22–1.48)	0.250		
Daily sleep hours	Less than 7 h	193 (84.6)	35 (15.4)	Ref.		Ref.	
	7–9 h	143 (92.3)	12 (7.7)	0.46 (0.23–0.92)	0.029*	0.52 (0.24–1.14)	0.105
	More than 9 h	19 (90.5)	2 (9.5)	0.58 (0.13–2.60)	0.477	0.94 (0.18–4.89)	0.938
Experienced food scarcity due to COVID-19	No	181 (94.8)	10 (5.2)	Ref.		Ref.	
	Yes	174 (81.7)	39 (18.3)	4.05 (1.96–8.37)	<0.001*	3.18 (1.29–7.86)	0.012*
Experienced any crime or domestic violence during the pandemic	No	313 (87.4)	45 (12.6)	Ref.			
	Yes	42 (91.3)	4 (8.7)	0.66 (0.23–1.94)	0.452	_	
Migrate or move to the home village during the pandemic	No	326 (89.6)	38 (10.4)	Ref.		Ref.	
	Yes	29 (72.5)	11 (27.5)	3.25 (1.51–7.03)	0.003*	3.26 (1.30–8.19)	0.012*
Have you received any support during the pandemic	No	196 (86.0)	32 (14.0)	Ref.		Ref.	
	Yes	159 (90.3)	17 (9.7)	0.65 (0.35–1.22)	0.184	0.63 (0.31–1.32)	0.222
Perception of safety in the neighborhood	Unsafe	69 (84.1)	13 (15.9)	Ref.			
	Safe	286 (88.8)	36 (11.2)	1.49 (0.75–2.97)	0.250	_	
Employment situation during the pandemic	No change	182 (87.9)	25 (12.1)	Ref.			
	Lost job/working hours reduced	173 (87.8)	24 (12.2)	1.01 (0.55–1.84)	0.974	_	
Unable to seek healthcare due to financial difficulties during pandemic	No	138 (90.8)	14 (9.2)	Ref.		Ref.	
	Yes	217 (86.1)	35 (13.9)	1.59 (0.83–3.06)	0.166	1.35 (0.55–3.36)	0.509
Household income status during the pandemic	Stayed the same	84 (83.2)	17 (16.8)	Ref.		Ref.	
	Decreased	271 (89.4)	32 (10.6)	0.58 (0.31–1.10)	0.097	0.57 (0.24–1.34)	0.197
Borrow money to meet daily needs during pandemic	No	106 (89.1)	13 (10.9)	Ref.			
	Yes	249 (87.4)	36 (12.6)	1.18 (0.60–2.31)	0.632	_	

*Significant at *p* < 0.05; ^CI^Confidence Interval, ^AOR^Adjusted Odds Ratio; ^OR^Odds Ratio; ^a^chronic illness such as cancer, heart disease, stroke, diabetes, kidney failure, arthritis; ^b^Modified Kuppuswamy Socioeconomic scale.

The study revealed that slum dwellers who earned between 7001 and 12000 BDT per month (OR2 = 0.38; 95% CI: [0.18–0.79]; *p* = 0.011) or more than 12,000 BDT per month (OR2 = 0.33; 95% CI: [0.16–0.68]; *p* = 0.003) were less likely to report PTSD symptoms compared to those who earned 7000 BDT or less per month.

Slum dwellers who sleep less than 7 h per day are more likely to experience PTSD symptoms than those who sleep 7–9 h (OR2 = 0.46; 95% CI: [0.23–0.92]; *p* = 0.029). Individuals living in urban slums who used unimproved or community latrines (OR2 = 4.15; 95% CI: [2.13–8.11]; *p* < 0.001) during the pandemic were four times more likely to experience PTSD symptoms compared to those who used latrines shared between two or more households or family latrines.

**Figure 2.**
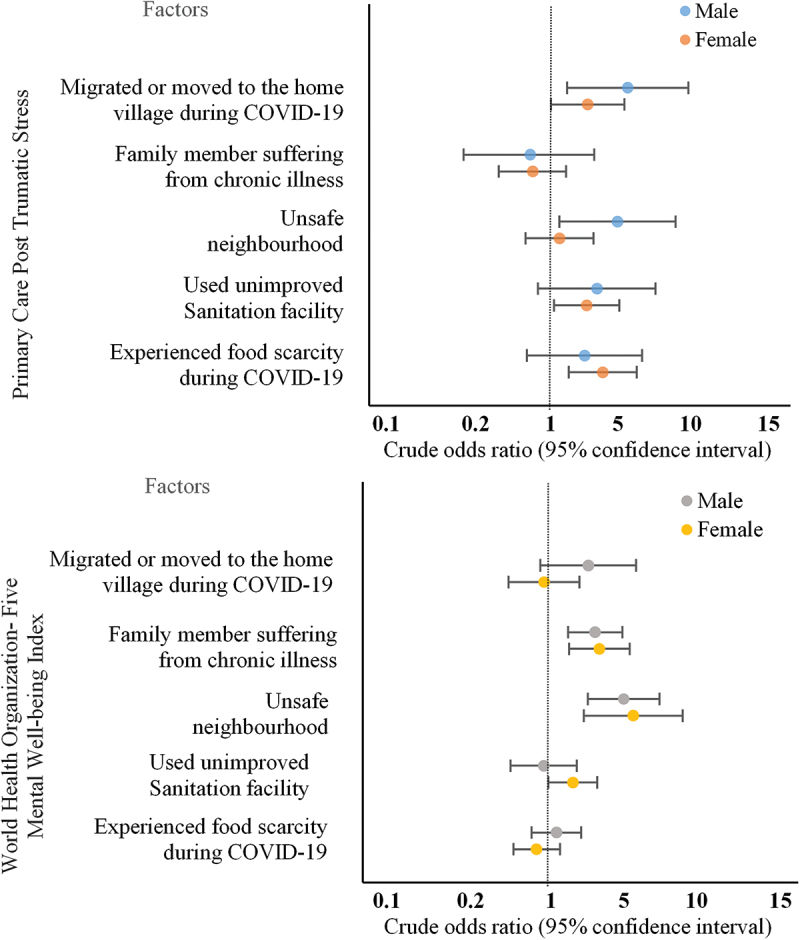
Models of association between factors and mental health problems (probable post-traumatic stress disorder and mental well-being index) of slum dwellers.

Slum dwellers who experienced food scarcity due to the pandemic were more likely to have PTSD symptoms in the aftermath of COVID-19 (AOR2 = 3.18; 95% CI: [1.29–7.86]; *p* = 0.012). The study also found that slum dwellers who migrated or moved to their home village during the pandemic (AOR2 = 3.26; 95% CI: [1.30–8.19]; *p* = 0.012) were more likely to have PTSD symptoms in the aftermath of COVID-19. Women are more likely to report symptoms of PTSD regarding food scarcity (OR2 = 3.79; 95% CI: [1.59–7.05]; *p* < 0.05), migration to the home village (OR2 = 2.59; 95% CI: [1.025–6.55]; *p* < 0.05) and use of unimproved sanitation (OR2 = 2.52; 95% CI: [1.10–5.77]; *p* < 0.05) during the pandemic (Figure 2). However, men were more likely to report symptoms of PTSD regarding unsafe neighborhood (OR2 = 5.13; 95% CI: [1.26–9.11]; *p* < 0.05) and migration to their home village (OR2 = 6.15; 95% CI: [1.53–10.34]; *p* < 0.05) during the pandemic.

## 4. Discussion

The study assessed the rate of anxiety and depressive symptoms of slum dwellers and found that approximately 50% of men and women residing in slums reported anxiety; the percentage is relatively high and consistent with prior research conducted among slum dwellers in Dhaka (Koly et al., 2021). While over one-fourth of men (29.5%) and one-third of women (35.1%) reported having depression symptoms, this percentage is unquestionably higher than the estimated 16.3% national weighted prevalence of depression among people living in urban and rural areas (Wahid et al., 2023).

Our study evaluated post-COVID mental health conditions among slum dwellers and revealed that overall 65.4% of females and 55.7% of males reported experiencing poor psychological health. Males and younger (25 years) slum dwellers were found to be less likely to experience poor mental well-being, which aligns with a prior study conducted among residents of Dhaka city slums (Gruebner et al., 2012). Moreover, our study revealed that the rate of PTSD symptoms was higher among female slum dwellers (18%) than their male counterparts (4.5%), with females being three times more likely to experience PTSD symptoms than males. The findings are consistent with prior studies that reported females are two to three times more susceptible to developing PTSD than males from stressful life experiences such as severe sickness, accidents or violence, or the loss of loved ones (Boyraz & Legros, 2020; Olff, 2017; van den Berg et al., 2017). Women are at a heightened risk of experiencing sexual assault, which is associated with an increased chance of developing PTSD (Tolin & Foa, 2006). Prior studies also revealed that after traumatic incidents, women are more likely to experience acute psychological disorders as well as recall incidents and acute stress responses than men (Kendler et al., 2001; Liu et al., 2020), which may partially explain the higher level of PTSD symptoms among women in our study. Furthermore, fluctuations in hormones on a monthly and lifetime basis may increase the likelihood of psychological disorders in women (Kuehner, 2017; Li & Graham, 2017); notably, elevated levels of progesterone during trauma exposure could play a role in the consolidation of memories related to that incident, which might lead to flashbacks of traumatic experiences, such as symptoms of PTSD (Li & Graham, 2017). Since female slum residents may have a higher risk of experiencing mental health problems, healthcare providers should monitor for psychological issues in the aftermath of any pandemic event. Mental health professionals collaborating with primary care providers can play an essential role in improving mental health conditions for older-aged and female slum dwellers while also mitigating the stigma around mental health disorders. Future research must prioritize longitudinal studies that monitor the mental health outcomes of impoverished older people over time while also examining the impact of mental health stigma on older and female slum residents.

Slum dwellers who lived a long time (more than 30 years) in the slum area were found to be two times more prone to experience poor mental well-being than those who lived less than 15 years in the slum area. One possible explanation is that individuals who have been raised and lived in urban slums for a prolonged period face serious challenges in obtaining basic necessities and the overcrowding that is prevalent in these areas can cause much stress among residents, which can have the potential to impact mental well-being. Moreover, in densely populated urban areas, poor living conditions and security might provoke unhealthy behaviors such as substance use, poor dietary choices and a lack of physical activity, all of which can negatively affect mental health (Gruebner et al., 2012; Zulu et al., 2011).

According to our research, Muslims in slums were found to be less likely to experience PTSD symptoms than non-Muslims. Previous studies conducted in Hong Kong (ethnic minorities from South Asia) and the UAE reported that Muslims experienced lower levels of stress, anxiety and depression and higher levels of positive religious coping during the pandemic (Thomas & Barbato, 2020; Wong et al., 2022). Muslims engage in more constructive religious coping mechanisms, such as viewing stressors as positive and seeking God’s love and care while also accepting crises and tragic occurrences as God’s wise plan (Mahamid & Bdier, 2021; Wong et al., 2022). Additionally, the Islamic faith dissuades individuals from self-harming practices and losing faith in God’s kindness in calamities (Abu-Raiya & Jamal, 2021; Mahamid & Bdier, 2021), which may partially explain the lower level of PTSD symptoms among Muslims in our study.

Our study also revealed that slum dwellers with low household income (≤7,000 BDT), no formal education, used unimproved or community latrines during the pandemic (particularly women) and short sleep duration (less than 7 h) were found to be more likely to experience PTSD symptoms. However, these findings showed no significant relationship with the adjusted model. Previous studies showed similar findings and reported that lower income, sleep duration, education and sanitation are associated with PTSD symptoms (Boyraz & Legros, 2020; Straus et al., 2022; Zhang et al., 2020). According to a previous study, women in particular may encounter challenges due to inadequate sanitation facilities, and they may fear or experience physical or sexual abuse when using unimproved sanitation (Kimutai et al., 2023). This situation could worsen during the pandemic, potentially impacting mental health and leading to heightened stress.

Our study revealed that those who lived with a chronically ill family member were at greater risk of experiencing poor mental well-being. These findings align with prior studies (Gruebner et al., 2012). Slum dwellers, who disclosed their neighborhood as safe, were less likely to face poor mental well-being than those who disclosed an unsafe neighborhood, consistent with a prior study, as an unsafe neighborhood is associated with poor health (Velasquez et al., 2021). Furthermore, studies have reported that safety in the neighborhood promotes beneficial impacts on mental well-being and in the framework of society (Gruebner et al., 2011; Mouratidis, 2020). Urban development initiatives should prioritize improving neighborhood safety and offering mental health support among slum dwellers to mitigate the psychological effects of residing in unsafe areas. Future research should evaluate the long-term psychological effects of living with a chronically ill family member and residing in unsafe neighborhoods.

In our research, we found that slum dwellers who experienced food scarcity during COVID-19 were found to be two times more likely to report PTSD symptoms in the aftermath of COVID-19. A prior study reported comparable findings that the COVID-19 pandemic led to food insecurity, which was linked to more severe symptoms of PTSD (Islam et al., 2021). Moreover, slum dwellers who borrowed money to meet their daily necessities during COVID-19 were more likely to report poor mental well-being, similar findings mentioned in prior research conducted among lower-income groups of people amid COVID-19 (Khan et al., 2024; Paul et al., 2021). Mental health professionals should be aware of the psychological impacts of financial stress and debt-related worries and can support their patients by connecting them to financial counseling or debt relief services that may assist in alleviating the financial load and its associated negative psychological consequences. Our study also revealed that slum dwellers who migrated to their home village during the pandemic are more likely to report PTSD, which aligns with previous research (Nasir et al., 2022). The fear of COVID-19 and the loss of temporary jobs forced the slum dwellers to migrate to their home villages for daily survival, which may have adversely affected their mental health conditions (Awasthi & Mehta, 2020; Khan et al., 2024; Sohel et al., 2022).

It is of utmost importance to address this issue and take initiatives to ensure that vulnerable individuals are not further marginalized during challenging times. Our study reports on PTSD symptoms among slum dwellers almost 2 years after the pandemic, providing valuable insight into the severity of the mental health effects of COVID. Despite its valuable findings, this study has several limitations, including recall bias and the inability to provide long-term trends. Our study only included a particular age group individual (17–70 years); future studies should also focus on the youngest and oldest individuals. We recommend future longitudinal studies to investigate the causal relationship between selected variables and poor mental health, particularly PTSD among women. Furthermore, we did not include the food habits of slum dwellers in our study; future longitudinal research should explore the long-term impact of mental health and food habits among slum residents in Bangladesh.

## 5. Conclusions

The findings of this study provide insight into the notable psychological challenges experienced by slum residents in the post-COVID context and various risk factors associated with poor psychological health outcomes in these communities. Individuals with lower income and education, less sleep, unimproved sanitation facilities and limited food access during the pandemic exhibited higher levels of PTSD symptoms in the aftermath of COVID-19. Uncertainties and limited access to essential resources have exacerbated mental health challenges, underscoring the importance of addressing the psychological repercussions of the pandemic alongside the physical health aspects. Furthermore, individuals who borrowed money to meet their daily needs and lived in unsafe neighborhoods experienced poor mental health, highlighting the importance of social support and financial aid in mitigating the negative impacts of the pandemic on mental health. The vulnerable population urgently needs targeted interventions such as skill-building/vocational training for long-term employment, neighborhood safety (e.g. controlling substance use, drug activity and violence), gender-segregated sanitation facilities and anti-stigma awareness programs to support their mental health. Collaboration with international organizations and NGOs can aid in training and facilitate the implementation of such initiatives.

## Supplementary Material

supplementary file 4.pdf

supplementary file 2_.pdf

supplementary file 1.tiff

supplementary file 3.tif
