# Supplementary material for: Mental health and post-traumatic stress among unprivileged people in the aftermath of COVID-19 pandemic in Southwest Bangladesh: a cross-sectional study
Source: Cogent Ment Health. 2025 Mar 26;4(1):2484006. doi: 10.1080/28324765.2025.2484006 (PMC12442989; doi:10.1080/28324765.2025.2484006)

**Appendix C:** Infographic

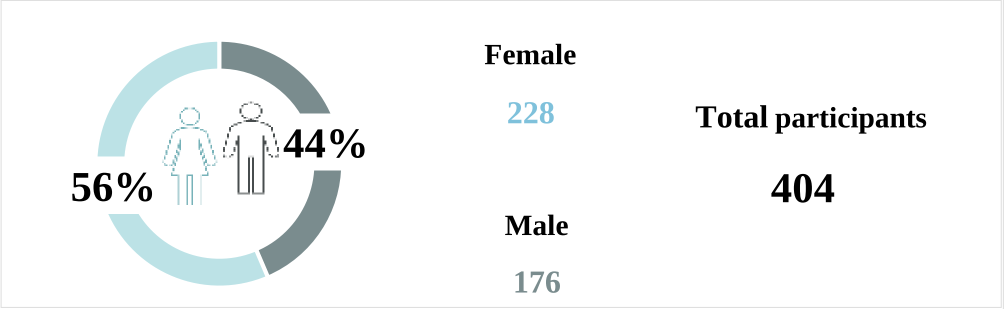


# KCC (Khulna City Corporation) Slum Infographics

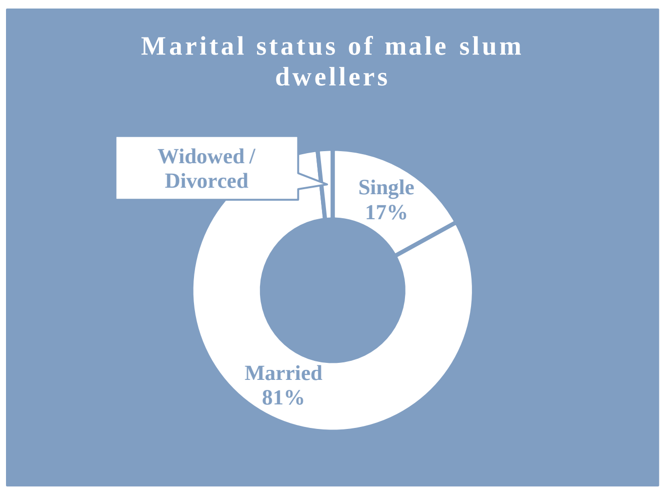


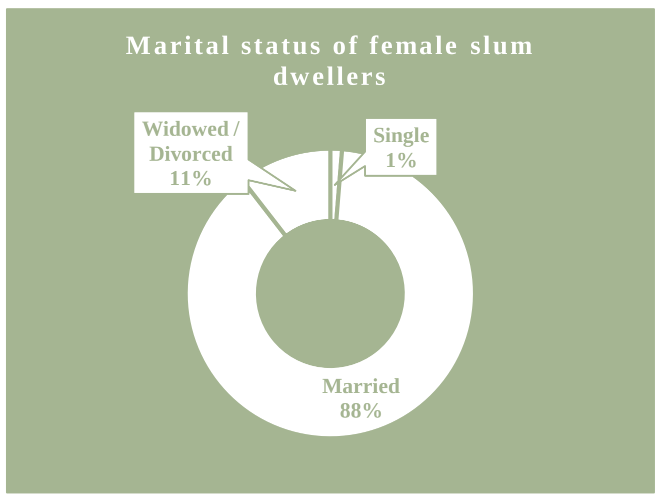


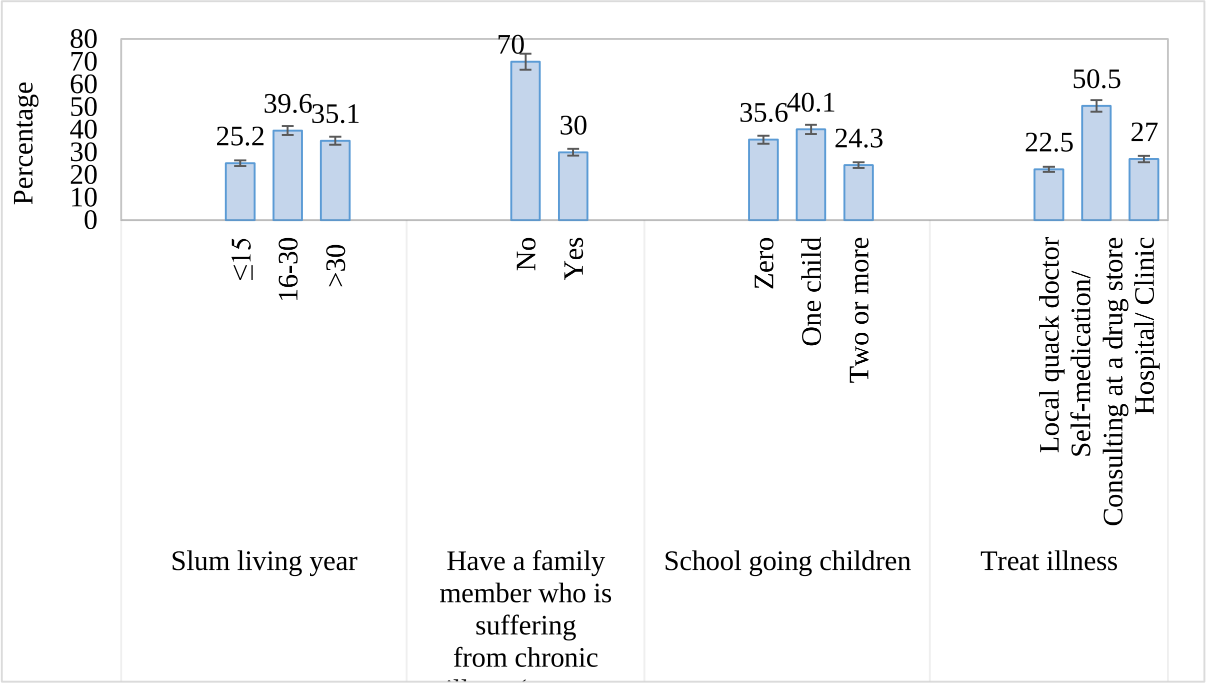


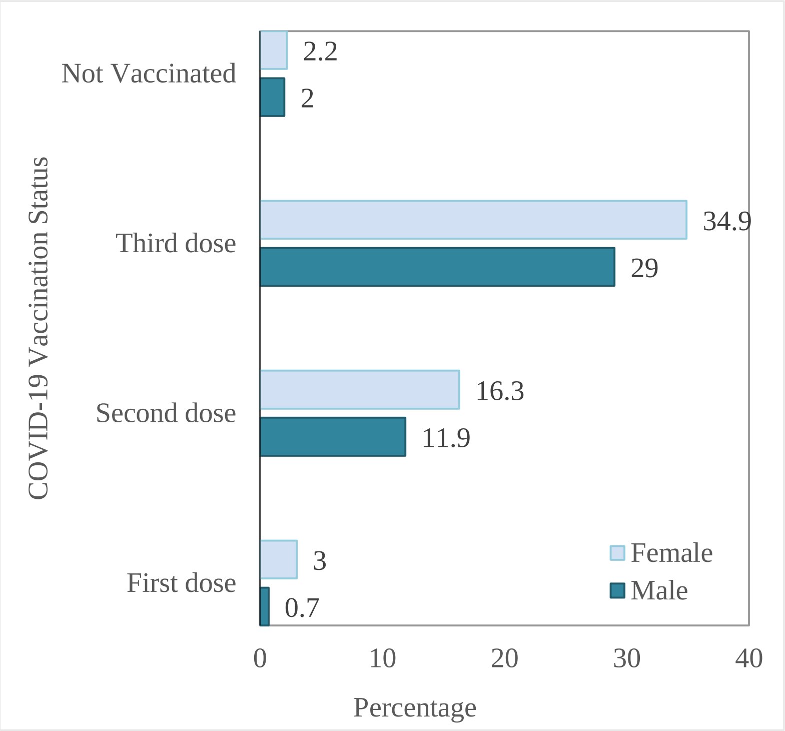


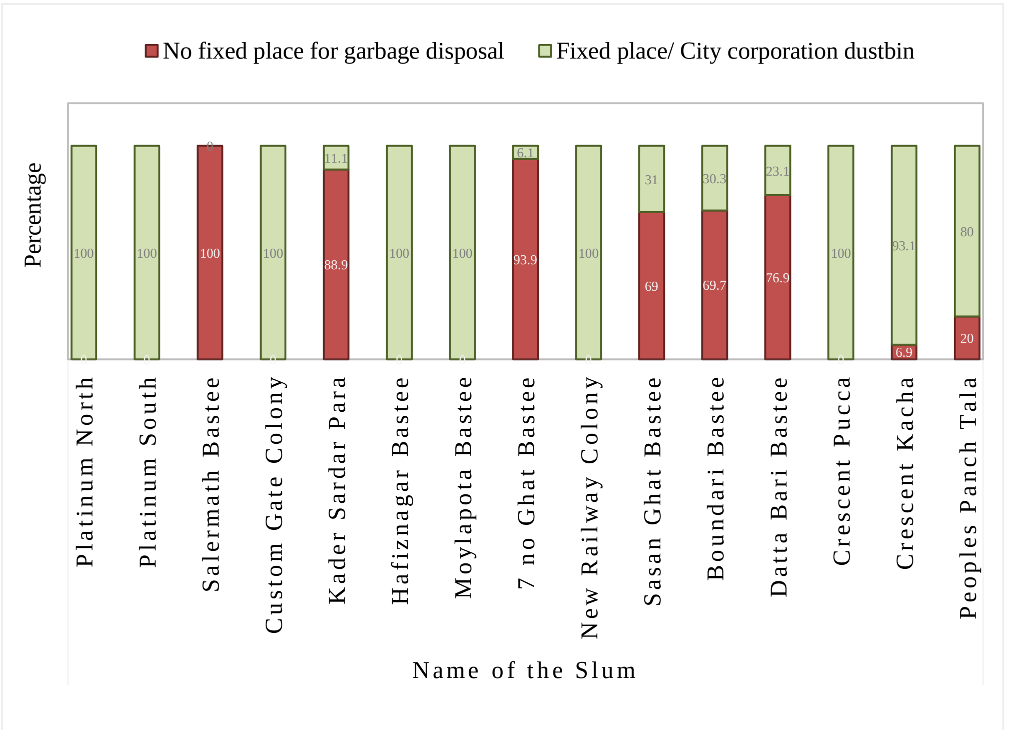


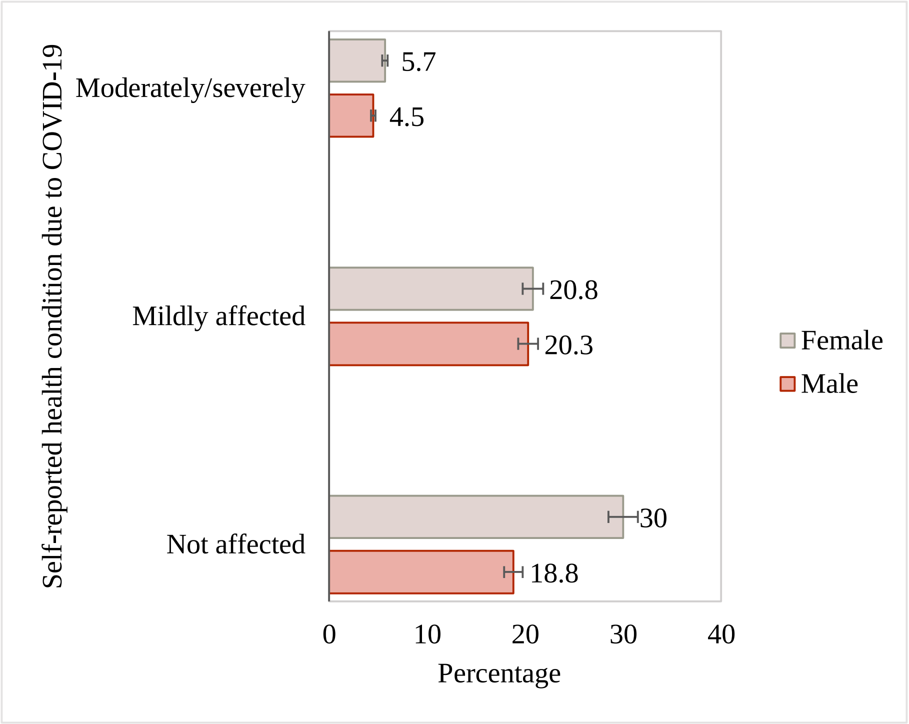


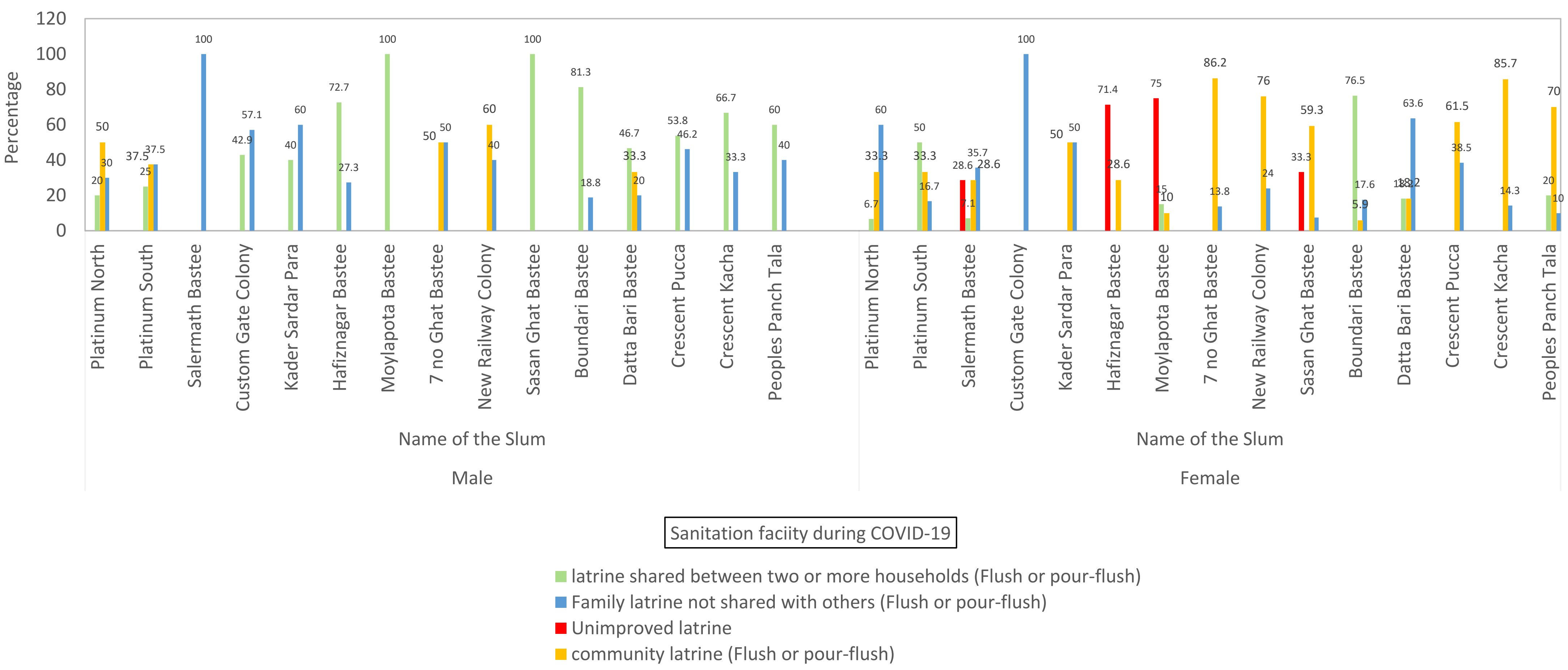


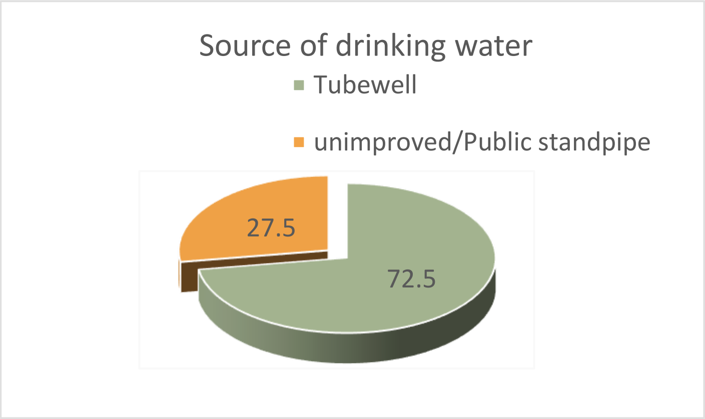


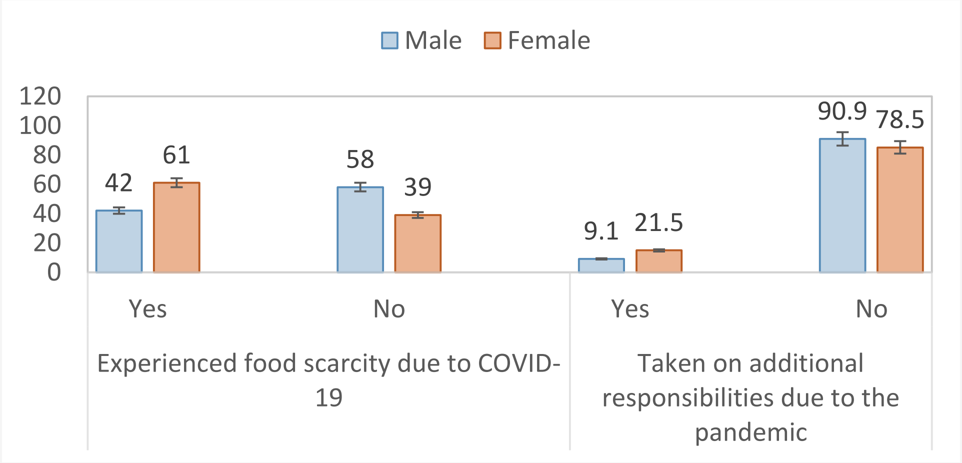


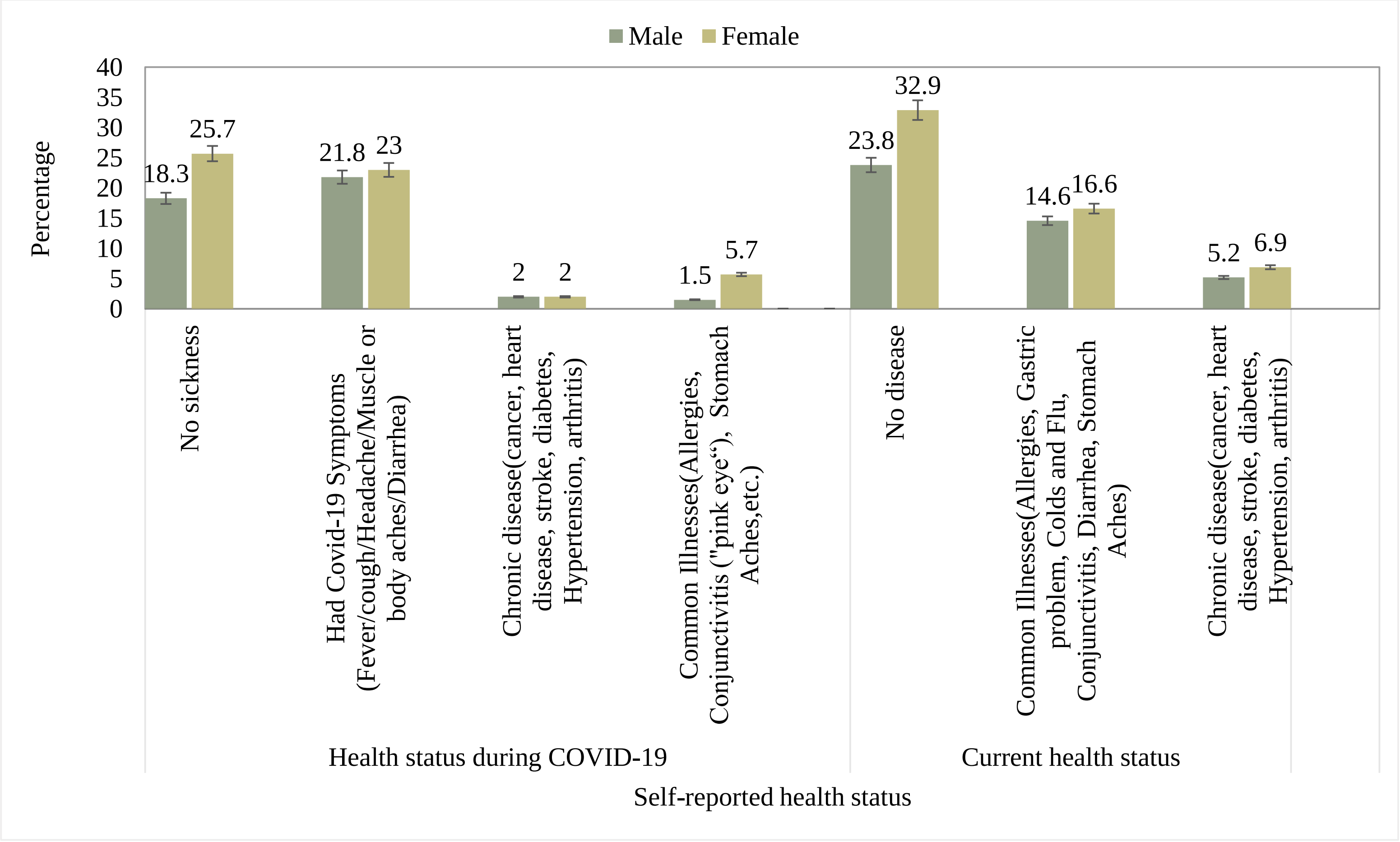

Supplement: supplementary file 4.pdf [file OAMH_A_2484006_SM6052.pdf]
