# Supplementary material for: Mental health and post-traumatic stress among unprivileged people in the aftermath of COVID-19 pandemic in Southwest Bangladesh: a cross-sectional study
Source: Cogent Ment Health. 2025 Mar 26;4(1):2484006. doi: 10.1080/28324765.2025.2484006 (PMC12442989; doi:10.1080/28324765.2025.2484006)
Supplement: supplementary file 2_.pdf [file OAMH_A_2484006_SM6051.pdf]

Table 1. Socioeconomic, demographic COVID-19 related characteristics of slum dwellers

**Variables**		**N (%)**	**Variables**		**N (%)**
Gender	Male	176 (43.56)	Religion	Other Religion	23 (5.69)
	Female	228 (56.44)		Muslim	381 (94.31)
Age (years)	≤25	96 (23.76)	Slum living year	≤15	102 (25.25)
	26-40	174 (43.07)		16-30	160 (39.60)
	> 40	134 (33.17)		> 30	142 (35.15)
Monthly household income (BDT	≤7000	78 (19.31)	Education level	No formal education	114 (28.22)
	7001-12000	148 (36.63)		Primary	207 (51.24)
	> 12000	178 (44.06)		Secondary/Higher	83 (20.54)
Family size	≤ 3	105 (25.99)	Family member suffering from chronic illness[a]	No	283 (70.05)
	4-5	197 (48.76)			
	> 5	102 (25.25)		Yes	121 (29.95)
SES[b] (Socioeconomic Status)	Extreme Poor	8 (1.98)	Sanitation facility during Pandemic	Latrine shared between two or more households /Family latrine	226 (55.94)
	Poor	292 (72.28)			
	Lower middle class	104 (25.74)		Unimproved/ Community latrine	178 (44.06)
Garbage disposal	No fixed place	163 (40.35)	Perception of safety in the neighborhood	Unsafe	82 (20.30)
	Fixed place/City corporation dustbin	241 (59.65)		Safe	322 (79.70)
Health status during COVID	Chronic/Common illness	45 (11.14)	Daily sleep hours	Less than 7 h	228 (56.44)
	COVID Symptoms	181 (44.80)		7-9 h	155 (38.37)
	No Sickness	178 (44.06)		More than 9 h	21 (5.20)
Experienced food scarcity due to COVID-19	No	191 (47.28)	Experienced any crime or domestic violence during the pandemic	No	358 (88.61)
	Yes	213 (52.72)		Yes	46 (11.39)
Migrate or move to the home village during the pandemic	No	364 (90.10)	Have you received any support during the pandemic	No	228 (56.44)
	Yes	40 (9.90)		Yes	176 (43.56)
Employment situation during the pandemic	No change	207 (51.24)	Unable to seek healthcare due to financial difficulties during pandemic	No	152 (37.62)
	Lost job/working hours reduced	197 (48.76)		Yes	252 (62.38)
Household income status during the pandemic	Stayed the same	101 (25.00)	Borrow money to meet daily needs during pandemic	No	119 (29.46)
	Decreased	303 (75.00)		Yes	285 (70.54)

[a] chronic illness-such as cancer, heart disease, stroke, diabetes, kidney failure, arthritis; [b] Modified Kuppuswamy Socioeconomic scale
