# Supplementary figures and images for: Mental health and post-traumatic stress among unprivileged people in the aftermath of COVID-19 pandemic in Southwest Bangladesh: a cross-sectional study
Source: Cogent Ment Health. 2025 Mar 26;4(1):2484006. doi: 10.1080/28324765.2025.2484006 (PMC12442989; doi:10.1080/28324765.2025.2484006)

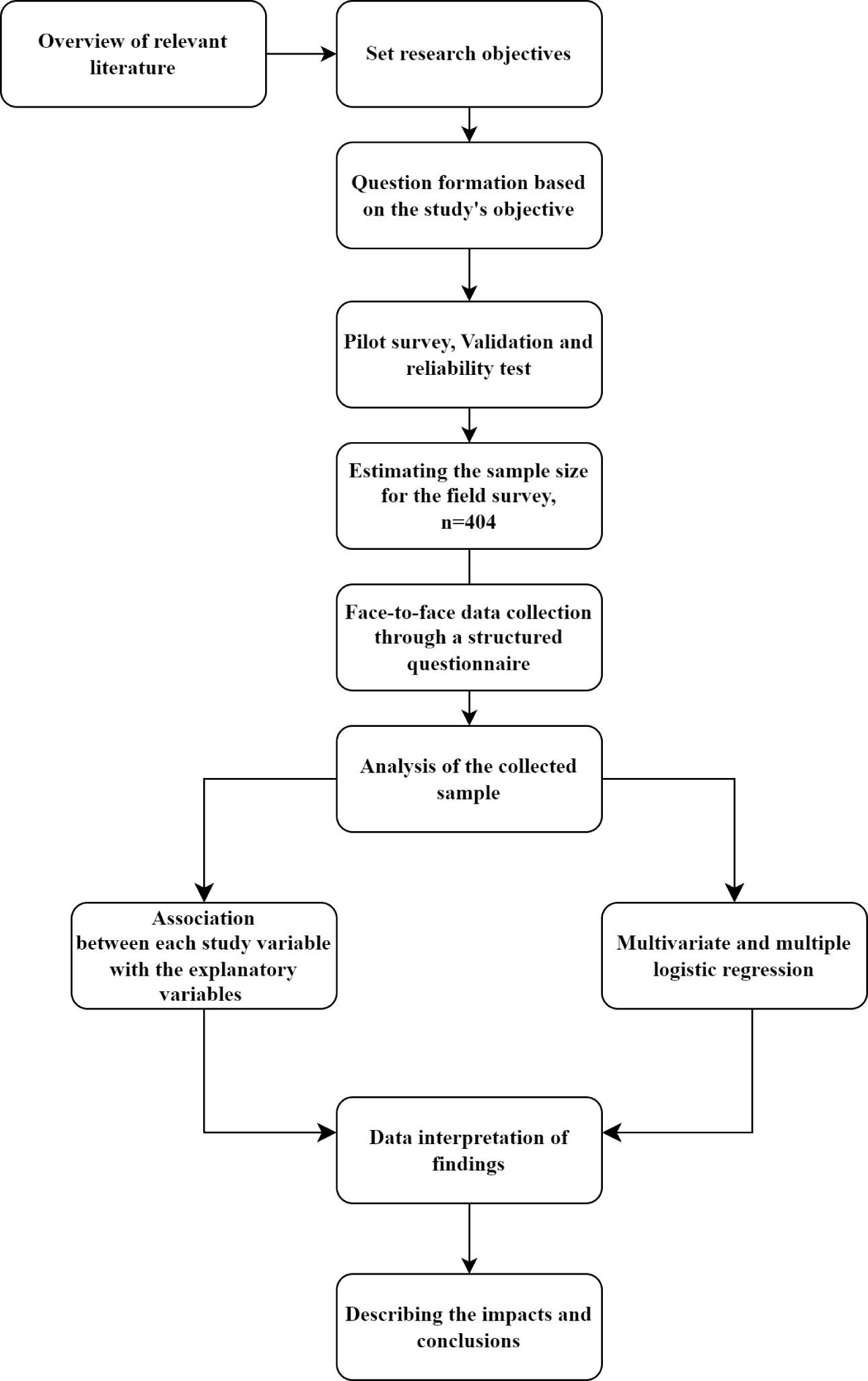

Supplement: supplementary file 1.tiff [file OAMH_A_2484006_SM6050.tiff]

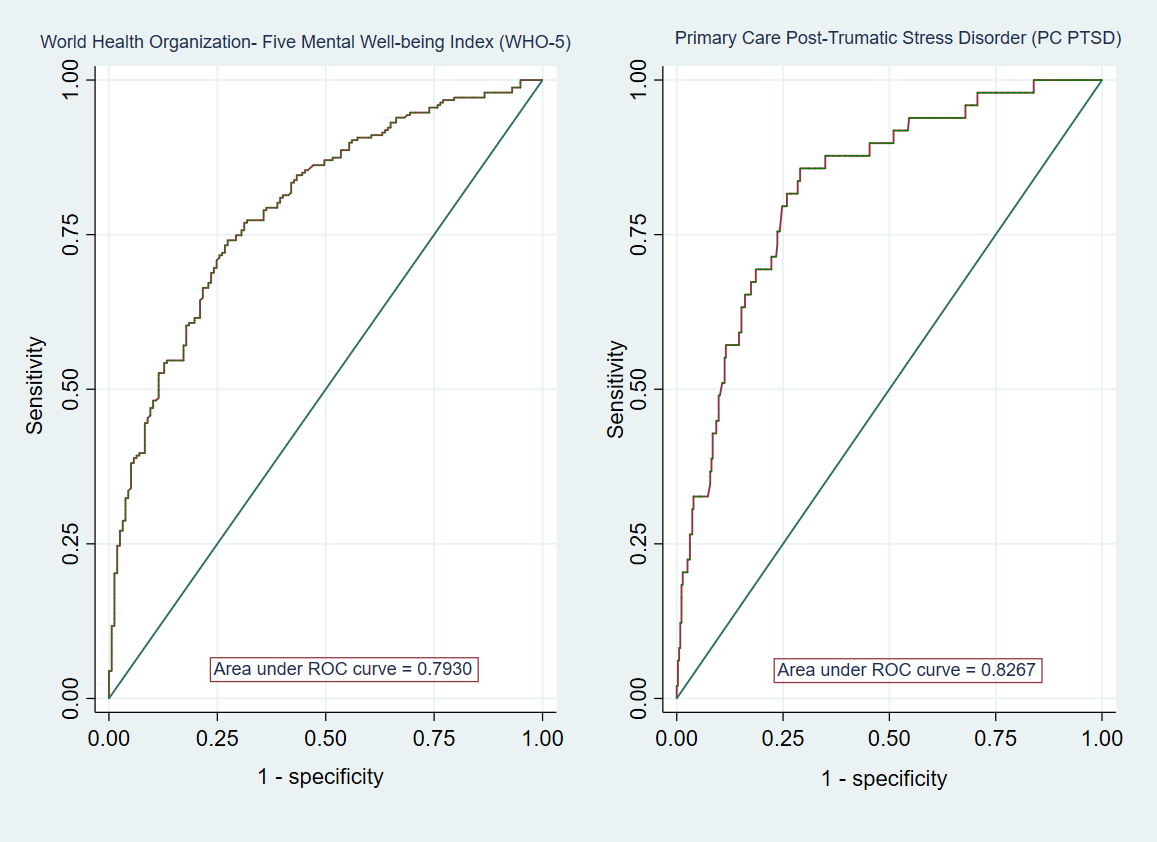

Supplement: supplementary file 3.tif [file OAMH_A_2484006_SM6049.tif]
